# Fracture of Inferior Maxillary Bone

**Published:** 1875-10

**Authors:** 


FRACTURE OF INFERIOR MAXILLARY BONE. By Jas. F. Montgomery, M.D., of Sacramento, Cal. Pamphlet; pp. 17.

